# Dynamics of positional warfare malaria: Finland and Korea compared

**DOI:** 10.1186/1475-2875-7-171

**Published:** 2008-09-08

**Authors:** Lena Huldén, Larry Huldén

**Affiliations:** 1 Department of Forest Ecology, Faculty of Agriculture and Forestry, University of Helsinki, Finland; 2 Finnish Museum of Natural History, University of Helsinki, Finland

## Abstract

**Background:**

A sudden outbreak of *vivax *malaria among Finnish troops in SE-Finland and along the front line in Hanko peninsula in the southwest occurred in 1941 during World War II. The common explanation has been an invasion of infective *Anopheles *mosquitoes from the Russian troops crossing the front line between Finland and Soviet Union. A revised explanation is presented based on recent studies of Finnish malaria.

**Methods:**

The exact start of the epidemic and the phenology of malaria cases among the Finnish soldiers were reanalyzed. The results were compared with the declining malaria in Finland. A comparison with a corresponding situation starting in the 1990's in Korea was performed.

**Results and discussion:**

The malaria cases occurred in July in 1941 when it was by far too early for infective mosquitoes to be present. The first *Anopheles *mosquitoes hatched at about the same time as the first malaria cases were observed among the Finnish soldiers. It takes about 3 – 6 weeks for the completion of the sporogony in Finland. The new explanation is that soldiers in war conditions were suddenly exposed to uninfected mosquitoes and those who still were carriers of hypnozoites developed relapses triggered by these mosquitoes. It is estimated that about 0.5% of the Finnish population still were carriers of hypnozoites in the 1940's. A corresponding outbreak of *vivax *malaria in Korea in the 1990's is similarly interpreted as relapses from activated hypnozoites among Korean soldiers.

The significance of the mosquito induced relapses is emphasized by two benefits for the *Plasmodium*. There is a synchronous increase of gametocytes when new mosquitoes emerge. It also enables meiotic recombination between different strains of the *Plasmodium*.

**Conclusion:**

The malaria peak during the positional warfare in the 1940's was a short outbreak during the last phase of declining indigenous malaria in Finland. The activation of hypnozoites among a large number of soldiers and subsequent medication contributed to diminishing the reservoir of malaria and speeded up the eradication of the Finnish malaria. A corresponding evolution of Korean malaria is anticipated with relaxed tensions and decreasing troop concentrations along the border between South and North Korea.

## Background

Indigenous *vivax *malaria was a common cause of death in the 18^th ^and 19^th ^century in Finland [1]. The decline of malaria commenced late in the 18^th ^century and malaria gradually decreased without countermeasures during the 19^th ^and 20^th ^century [2]. Medication was sparingly used. At the beginning of the 1930's there were fewer than 10 cases annually and the last cases of indigenous malaria were documented in 1953 and 1954 [3].

**Figure 1:**
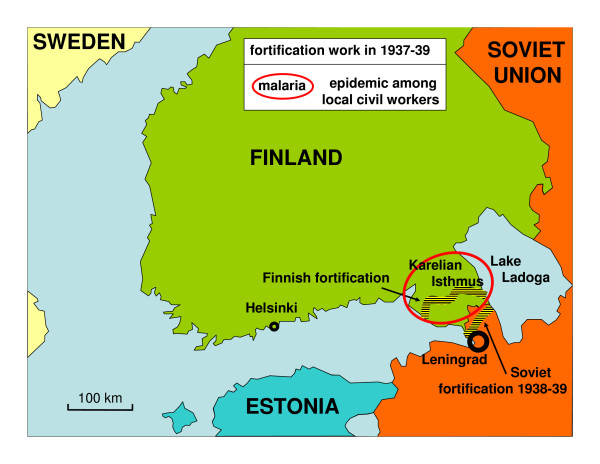
Military situation in Finland before the Winter War 1939–40.

**Figure 2:**
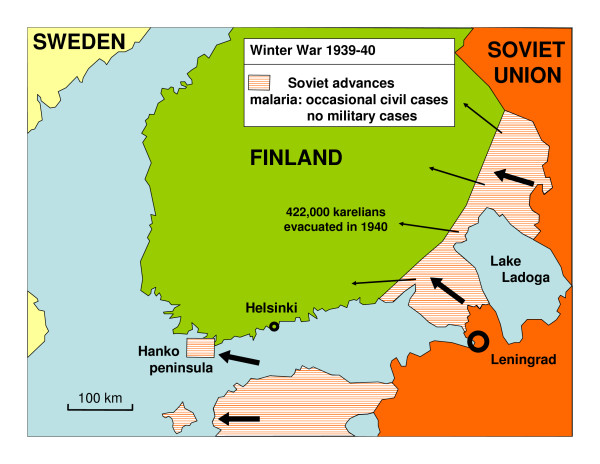
**Military situation after the Winter War in 1940.** Over 400,000 karelians were evacuated from the occupied area. There were no cases of malaria among military troops.

**Figure 3:**
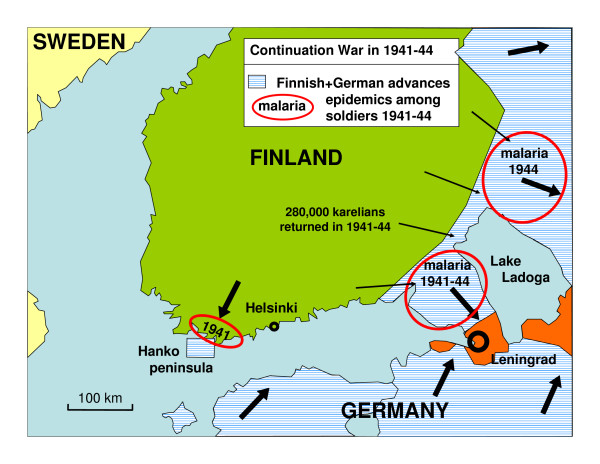
**Finnish and German maximum advances during Continuation War in 1941–44 in the surroundings of Finland.** Malaria occurred among Finnish troops along three frontlines: Hanko peninsula, the Karelian Isthmus and East Karelia east of Lake Ladoga. During the war 280,000 Karelians returned to their homes but there were very few cases of malaria among civilians.

**Figure 4:**
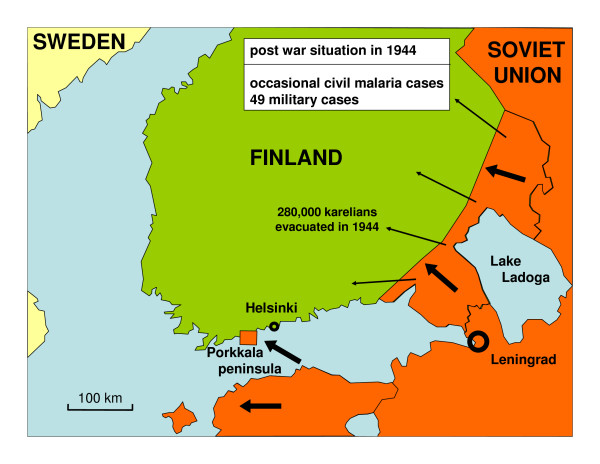
**Post war Finland in 1944.** The Karelians were again evacuated from Karelia. There were very few cases of malaria among civilians.

During World War II Finland fought two wars with the Soviet Union. The Winter War lasted from November 1939 to March 1940. Allied with Germany, Finland started the Continuation War in July 1941. Finnish troops invaded large parts of Soviet Karelia. In January 1942 the front line became more immobile and the positional warfare lasted to June 1944 [4,5]. The war was lost in September 1944. During the war recurrent malaria epidemic became an increasing problem among Finnish soldiers. The malaria situation among the Soviet and German soldiers is not known. The Finnish malaria epidemic in 1941–1945 has been studied in detail by Hernberg [6-8]. He proposed the widely accepted explanation that infective mosquitoes spread malaria over the front line from Soviet soldiers. The main military events in relation to malaria in southern Finland are presented in Figures 1, 2, 3 and 4.

A corresponding positional warfare malaria epidemic commenced in the 1990's in the western part of the demilitarized zone (DMZ) between South and North Korea [9,10]. At the beginning of the 20^th ^century *vivax *malaria was common in Korea. During the Korean War (1950–53) there was a peak in malaria among both civilians and soldiers. Malaria declined rapidly in the 1960's and 1970's [11]. Both countries were declared officially free of malaria in 1979 [12]. Malaria re-emerged in South Korea in 1993 when one soldier got malaria near the DMZ. Later malaria increased rapidly peaking in 2000 when thousands of people, mostly soldiers, got malaria close to the DMZ. It also spread southwards in South Korea. There was a simultaneous epidemic of *vivax *malaria starting in 1995 in North Korea close to the DMZ [9]. The number of malaria cases in North Korea reached nearly 300,000 in 2001. The prevalent opinion is that malaria re-emerged in South Korea because of a continuous influx of infective mosquitoes from North Korea where malaria is expected to have persisted [10,13]. Detailed maps of the evolution of the recent epidemic in South Korea have been published [10,11].

In both Finland and Korea the malaria epidemics started in the military forces and only later spread among civilians. The data from Finland and Korea suggest that the key to understanding these epidemics is strongly connected with some special features of military activities. This study aims at resolving the origin of re-emerging malaria among soldiers in positional warfare conditions.

## Methods

Statistics on monthly war malaria in Finland in 1941–1945 was taken from Hernberg's studies [6-8]. Annual malaria cases in Finland in 1860–1954 were collected from several publications [2,3,14]. The military preparations in 1941 in Finland are quantitatively quite well known and they are used to explain the dynamics of the malaria epidemics during 1941–1945 [4,5]. Statistics on Korean malaria was taken from various sources [9-12]. The Korean military statistics, however, are less known because of the international tensions. United States sources were used to interpret recent changes in North Korean military strategy [15]. The estimation of the troop strengths near DMZ in South Korea and North Korea during the epidemics was indirectly interpreted according to the statistics on malaria prophylaxis [10]. Statistics on urbanization trends in the three countries was collected from scientific or official sources [16-19]. The criteria for urban and rural settlements varies between countries, but for the present study only the varying steepness of the urbanization rate is relevant independently of particular values.

## Results and discussion

### Finland 1941–1945

Finland mobilized 610,000 people in the armed forces in the Continuation War in 1941 and concentrated some 310,000 men along the front line with Soviet Union in Karelia in the southeast and on Hanko peninsula by the Gulf of Finland (ceded to Soviet Union after the Winter War in 1939–1940). The allied German troops (about 200,000 soldiers) were concentrated in the northern part of Finland [2,3].

**Figure 5:**
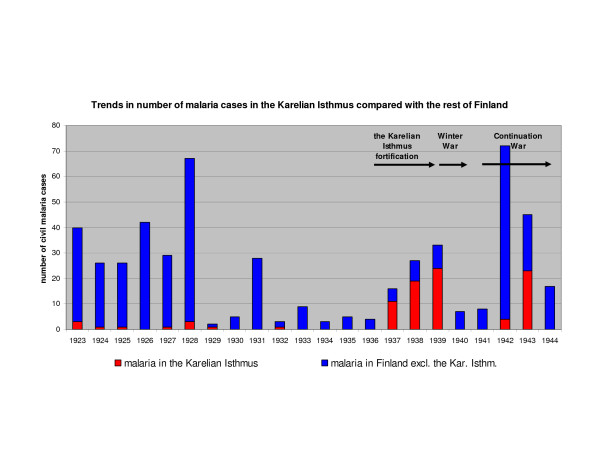
**Civil cases of malaria in 1923–44 in Finland.** The Karelian Isthmus and the rest of Finland are compared. Malaria cases had disappeared in the Karelian Isthmus in the beginning of 1930's. The cases in 1937–39 were mosquito triggered relapses. Most of the civil cases in 1942–44 are expected to be soldiers on leave [6].

**Figure 6:**
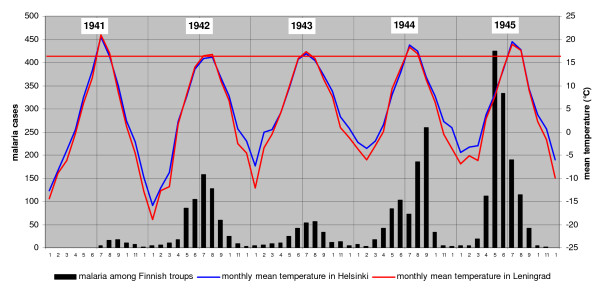
**Monthly military cases of malaria form 1941 to 1944 and civil cases in 1945.** The red horizontal line indicate the threshold temperature for sporogony of *Plasmodium vivax *in South Finland. Summer peaks of malaria cannot be explained by primary infections because *Anopheles messeae *hatch on average only in the latter half of July [1,19].

Annual civil malaria cases in Finland and the Karelian Isthmus from 1923 until the end of Continuation War in 1944 is presented in Figure 5. It is notable that malaria had almost disappeared from the Karelian Isthmus during the 1930's but suddenly increased in 1937 before the Winter War. In 1940 and 1941 there are no documented civil cases in that area. Already in July 1941 several malaria cases appeared among Finnish soldiers along both southern frontlines. Monthly military cases of malaria in 1941–1944 and civil cases after the war in 1945 are presented in Figure 6. Hernberg [5] studied the situation from 1941 to 1945 and used his statistics as a proof of existence of a dormant stage in humans. He concluded that Finnish soldiers at the beginning of the 1940's must have got the infection from infective mosquitoes flying across the front line from the Soviet positions because indigenous malaria was very rare in Finland at that time.

According to recent studies on historical malaria in Finland, primary infections occur only indoors from mid winter to spring and infective *Anopheles *mosquitoes do not exist in summer [1,19]. The *Anopheles *mosquitoes reach the adult stage in the latter half of July and August. In 1941 the mean temperature of June was cold (about 11°C) and July was warm (about 21°C). As a consequence, *Anopheles *mosquitoes had certainly hatched already in July, but they cannot then have been infective with sporozoites. Because of the low temperature in August the sporogony was slow.

Because the primary infections of malaria during the summer of 1941 are excluded the early malaria cases can only have been relapses from activated hypnozoites. Relapses are principally expected to be triggered by the bites of mosquitoes in natural conditions [19]. Hypnozoites can remain viable in the human liver at least nine years but probably a much longer time [19]. The main issue is to explain the source of hypnozoite carriers along the front line.

The basic cause of decline of malaria in Finland is now expected to have been gradual decrease of household size since the latter half of 18^th ^century from about ten to four members [20]. The household size of four members seems to be a threshold value for prevalence of malaria. The declining household size has a very high correlation with declining malaria frequency (p < 0.001). According to the Finnish statistics malaria disappeared when the household size decreased below four members in the 1950's. Likewise the European malaria declined in step with declining household size until it disappeared when household size passed below four members in all countries [21,22]. The mechanism of the decline, however, will be discussed in a separate paper.

The increasing level of urbanization is another factor that must be considered when interpreting the occurrence of relapses. If we accept that *Anopheles *mosquitoes are the principal triggers of relapses, then relapses should occur in situations where mosquitoes and humans meet. This happened in the rural Finland each year when the new generation of *Anopheles *hatched in July. The rural population decreased gradually when people moved into cities to look for better job opportunities. During the last phase of malaria in the 1920's and 1930's many people were still hypnozoite carriers when they moved into cities. People living in urban conditions in multi-storey buildings are rarely exposed to mosquitoes compared with people in rural conditions. A conclusion is that a certain fraction of the Finnish urban population in the late 1930's still had dormant hypnozoites in their liver. A steep decline of malaria frequency occurred in urban settlements about 20 years later than in rural settlements. Although the number of malaria cases was declining, the number of people carrying hypnozoites was still high enough for a new epidemic during suitable conditions. A suitable condition develops when a large random sample of the population congregates and is stationary for a considerable time. An occasional relapse may be triggered immediately but an epidemic requires a longer time for the new cycles of malaria to develop. Typical situations are positional warfare, refugee camps and large scale public work with provisional lodging during times of unemployment and depression (corresponding to "frontier malaria" in the 19^th ^century United States).

Most of the Finnish malaria cases since the late 1920's were probably relapses. The peak in malaria cases in 1931 is explained by depression and public work. The workers lived in temporary quarters in primitive conditions and were exposed to nocturnal mosquitoes. A major part of the malaria cases in 1937–1939 was clustered within the Karelian Isthmus during intensified fortification work before the war in November 1939. The military actions during the Winter War 1939–1940, however, did not contribute to the malaria cases because of the highly mobile front lines during the cold months. The soldiers slept in a tent or in a lean-to with temperatures down to -40°C. In such conditions mosquitoes cannot be active.

**Figure 7:**
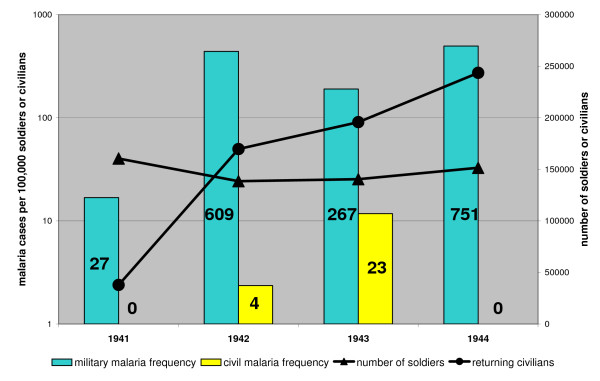
**Proportion of military and civil cases of malaria in the Karelian Isthmus. **The absolute numbers are given inside the bars. Most of the civil cases are expected to represent soldiers on leave [6].

During the Continuation War, the situation changed. Positional warfare conditions prevailed from January 1940 to June 1944. The soldiers slept in primitive dugouts and barracks (6–24 people in the same space) and remained stationary and continuously exposed to mosquitoes which over wintered together with humans. Because the sleeping places were heated the over wintering mosquitoes were forced to take blood during winter. Any malaria carrier would then have got a relapse triggered by the mosquitoes. Exactly in these conditions the malaria epidemic commenced. The first outbreak revealed in the summer in 1941 that hypnozoite carriers among the soldiers were able to initiate an epidemic. It is worth noting that there were very few civil cases on the Karelian Isthmus during the Continuation War although 280,000 civilians returned to their homes [Figure 7].

**Figure 8:**
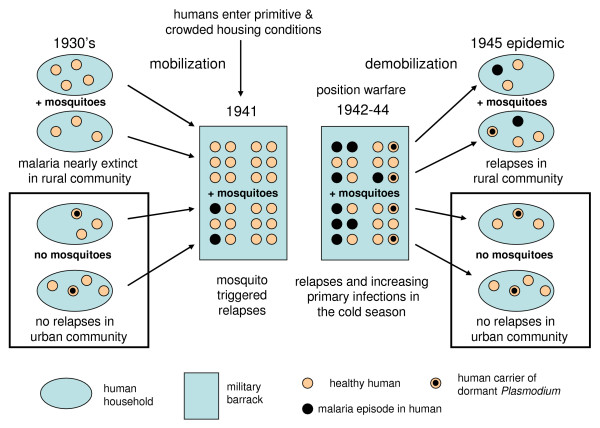
**Scheme of re-emerging malaria during the Continuation War.** In crowded conditions with mosquitoes the malaria epidemic starts with mosquito triggered relapses in hypnozoite carriers. In the next step mosquitoes become infective and gradually spread sporozoites into healthy humans. After the war hypnozoite carriers got relapses if *Anopheles *mosquitoes were present.

According to Hernberg [6] there were about 3000 cases of malaria among the Finns during 1941–1945. Because he did not know the true dynamics of relapses his judgement about separating primary infections from relapses cannot be relied on. A crude estimate of 1500 primary infections and 1500 relapses is accepted in this study. About 310,000 men were concentrated along the front line and exposed to mosquitoes. It can therefore be estimated that about 0.5% of the Finnish population still had hidden dormant hypnozoites in their liver in the 1940's. In 1945 after the war 1551 cases of malaria were reported all over Finland from February to October peaking in May and June. 299 patients had malaria two or several times so the remaining 1252 cases represent the true number of malaria infected persons. A detailed study on 868 patients revealed only 12 women. Practically all of them represented repatriated soldiers. Probably nearly all of the cases were relapses. They originated from primary infections that must have taken place during the war in the spring 1944 when many soldiers lived together in primitive conditions. The mosquitoes were then able to transmit malaria between infected and uninfected soldiers during the transmission season from December to May inside the dugouts and barracks. When the infected soldiers were repatriated after the war only those soldiers returning to conditions where they were locally exposed to mosquitoes got relapses. Those soldiers that returned to housing conditions without mosquitoes could still have hidden dormant stages of malaria in their liver a long time after the war. The schema in Figure 8 presents the principal mechanism of the outbreak of malaria in positional warfare conditions.

The key factor for the re-emergence of malaria is the number of people sleeping in the same space. The average number of soldiers sleeping in the same space is usually much higher than the empirically found threshold value of four humans per room. The success of *Plasmodium *is linked to interactions between groups of humans. If the groups are too small with two or three humans, the groups become more isolated and the *Plasmodium *faces a higher extinction rate than the re-colonization rate. If the group size is more than four humans, the *Plasmodium *can gradually spread among humans. Principally malaria epidemics among military forces could be avoided if each soldier sleeps alone in a separate space like a room, a tent or a bed net. In such situations occasional malaria cases will always be isolated and more easily controlled than if a large number of people get malaria simultaneously.

Of special interest in the outbreak of malaria among Finnish troops is that it did not happen in fight situations, it was always behind the immediate front line. Because most *Anopheles *species are night biters (like *A. messeae *in Finland) they usually bite when people are sleeping. The behaviour of an infective mosquito is altered by the *Plasmodium*. Sporozoites in the salivary glands decrease the production of anti-coagulants and vasodilators by the mosquito which in effect forces the mosquito to bite repeatedly to get blood [19]. Another effect is that the chance of the bite getting unnoticed by the victim increases as human skin does not react on the decreased load of alien proteins. That means that all people sleeping in the same space could quickly get malaria by only one infective mosquito. All these circumstances points to a basically indoor transmission pattern of malaria in general.

Malaria has sometimes been associated with famine and hardship. During 1749 – 1849 there were 27 years associated with famine because of extreme weather conditions (floods, frost or drought with thousands of deaths) in Finland [23]. The mean annual number of deaths from malaria was 95.24 for the whole period and 103.07 for the 27 famine years. The difference is statistically insignificant. As a consequence the effect of famine or other hardship on malaria prevalence is expected to be irrelevant.

### Korea 1993–2006

There are several similarities in the evolution of malaria in Korea and Finland. *Plasmodium vivax *with long incubation time is common to both regions. During the Korean War in 1950–54, there was a high rate of malaria among all military forces. Later it declined in step with decreasing household size and increased urbanization. The average household size in South Korea reached four members in the 1980's. In North Korea, the household size was still higher in the 1990's, varying from 4.2 to 4.7 depending on sources [24].

Malaria was officially eradicated in both North Korea and South Korea in 1979 [12]. But at least in South Korea occasional cases, which were considered relapses after a long incubation, were still reported until 1984 [25]. Re-emergence of malaria commenced in 1993 in South Korea [10] and in 1995 in North Korea [9]. The number of cases in North Korea has been nearly 100 times higher than in South Korea. The malaria trend is now reversed in both countries by means of large scale national and international (especially in North Korea) counter measures.

The re-emergence of malaria has received much attention in South Korea. It is generally believed that it was a result of influx of infective *Anopheles sinensis *mosquitoes (the main vector) from North Korea [11,23]. This explanation is inconsistent with the fact that during the main transmission season in the summer, the prevailing wind direction comes from the south [26,27]. Unfed females of the vector species, *Anopheles sinensis*, can fly quite long distances, up to 9–12 km [28], but after a blood feed it remains 2–3 days in a safe place until egg-laying [29,30]. After the first egg batch, the female returns within 24 hours to a host to the next blood meal [31]. Infective mosquitoes have been collected at very low frequency in Korea, 0.01–0.06% of studied samples [23]. It has been shown that *A. sinensis *females are highly zoophilic and prefer cattle [29,30]. They take, however, readily blood from humans if cattle are not available [29]. It is also striking that malaria foci in South Korea in the 1960's were relatively stationary and malaria did not spread in neighbouring regions by means of flying mosquitoes [11]. The South Korean troops near DMZ had about a 6–7 time higher malaria incidence rate than the United States troops stationed in the same region in 1993–2005 [10]. That large difference in the incidence rate would not be expected if infective mosquitoes at random would bite soldiers close to DMZ. Considering the sum of all these factors, large scale spreading of malaria by mosquitoes from North Korea seems unlikely.

The behaviour of an infective *A. sinensis *is probably altered in the same way as *A. messeae *in Finland. The production of anticoagulants and vasodilators are reduced because of sporozoites in the salivary glands [19]. An infective mosquito female is forced to take repeated blood meals and will not fly far away from the blood source. A plausible explanation is that infected humans themselves are spreading malaria and the role of the mosquito is to locally transfer malaria between human individuals. In this way, *A. sinensis *can be regarded a good vector although the proportion of infective individuals is very low.

Re-emergence of malaria in Korea in the 1990's can be explained in a similar way as the epidemic during positional warfare malaria in Finland in the 1940's: hidden dormant stages in the liver of a small fraction of the Korean population. In the case of North Korea the strength of military forces increased from 288,000 to 665,000 within 100 km of the DMZ from 1980 to 1994 [15]. The number of soldiers close to the DMZ during the recent malaria epidemic has been at least 350,000 based on statistics on malaria prophylaxis [10]. In retrospect it is very likely that occasional relapses of *vivax *malaria still occurred in the 1980's and beginning of 1990's in North Korea corresponding to the South Korean cases in the 1980's. The increased military forces close to the DMZ created a situation resembling that in Finland in 1941. An increasing part of the urban population was mobilized in conditions with higher exposure to mosquitoes. The occasional carriers of hypnozoites developed relapses leading to a regional epidemic among military forces. This is in short the origin of the re-emergence of malaria in North Korea. To maintain parity in the military balance South Korea probably intensified the guarding of DMZ although public reports are not readily available. It is, however, known that more than 200,000 South Korean soldiers in the risk zone near DMZ got malaria prophylaxis in 2005 [10]. Increased number of soldiers close to the DMZ created a suitable situation for the re-emergence of malaria because viable hypnozoites presumably still occurred in occasional soldiers along DMZ in South Korea.

The higher magnitude of malaria cases in North Korea compared with South Korea is explained by a combination of several factors. The household size in North Korea is obviously higher than in South Korea as mentioned above. There was a higher proportion of urban hypnozoite carriers in North Korea than in South Korea because of a more rapid urbanization rate in North Korea which caused an apparent rapid decline of manifested malaria in 1970's. Rapid urbanization enabled a higher proportion of the human population to escape exposure to *Anopheles *mosquitoes. During the mobilization of more troops close to DMZ, a higher proportion of the civil population became again exposed to mosquitoes and caused a rapid epidemic (although not immediately reported). North Korea was badly prepared to withstand a malaria epidemic compared with South Korea because of many political and economic reasons.

**Figure 9:**
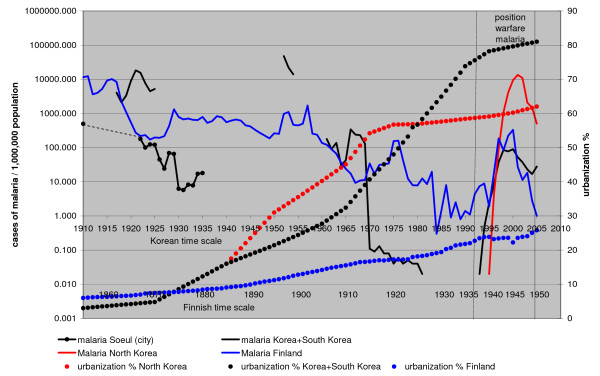
**Trench warfare malaria in Korea and Finland. **Time scale for Finland has been moved 55 years forwards. Note: The population of South Korea is about 10 times that of Finland and the lowest absolute values for Finland were about the same as those for Korea. On the logarithmic scale 1 for Finland and 0.1 for Korea both represent 4 cases of malaria. The malaria situation in Finland was only marginally different from that in Korea when a new epidemic commenced. The pronounced decline of malaria in Korea should be compared with the urbanization rate.

The effect of the difference in urbanization rates on malaria decline is illustrated when Finland and Korea are compared (Figure 9, data on urbanization from [29-31]). Urbanization level in Finland was still very low during the decline of malaria in the 1920's and 1930's. Malaria declined more slowly in Finland and the positional warfare malaria produced a broader peak than in Korea. In Finland malaria disappeared within ten years after World War II.

### The benefit of induced relapses for *Plasmodium*

The significance of the mosquito induced relapses is obvious. The timing of activation of a dormant stage in malaria is closely correlated with the emergence of the new generation of adult *Anopheles *mosquitoes as demonstrated for *vivax *malaria in the case of Finland [19]. The *Plasmodium *can optimize the production of gametocytes during the peak of mosquito activity. This leads to an efficient production of sporozoites for transmissions to new human hosts. If there are big clusters of people present, as in positional warfare conditions, there will be a rapid spreading of *Plasmodium *among humans.

The basic function of a mosquito induced relapse probably is that it enables meiotic recombination in mosquitoes. A human can have several primary infections without the activation of all hypnozoites. Each primary infection will increase the number of hypnozoites in the liver. When the hypnozoites are activated during the next mosquito season, there will be a synchronous development of gametocytes of different strains. This optimizes the possibility for a meiotic recombination within mosquitoes. Regular occurrences of meiotic recombination among a large human population will increase the adaptability of the *Plasmodium *to various *Anopheles *species and to variable seasonal and environmental conditions like those in Finland and Korea.

## Conclusion

The new interpretation of re-emerging *vivax *malaria in positional warfare conditions is based on two independent discoveries on Finnish malaria. Firstly, malaria transmission is mainly an indoor phenomenon. High correlation of a threshold value of four persons in household size and malaria prevalence points to the indoor transmission of malaria. The overall decline of household size below four persons and disappearance of malaria in Europe in the 20^th ^century strongly corroborates a universal indoor transmission independently of vector species and *Plasmodium *species. When any vector species becomes infective the *Plasmodium *alters the vector in the same direction. The vector is forced to repeatedly probe for blood and to remain close to humans. Outdoor transmissions of malaria are not excluded, but they have small impact on the long term statistics.

Secondly, the mosquito triggered relapse explains the exact timing of the activation of hypnozoites and presence of mosquitoes. Hypnozoites of *P*. *vivax *malaria may be viable for decades in human liver. This fact explains the re-emergence of malaria when it seemed to have been eradicated in recent decades. *Vivax *malaria is often described as unstable malaria. Actually, it is stable malaria but well adapted to fluctuating transmission opportunities.

Some principal conclusions from the study can be drawn. The primary means of the spreading of *Plasmodium vivax *is by humans and only secondary by the mosquitoes. The *Plasmodium *spends only a short time of its lifetime in the mosquito based on the relative length of sporogony. The time in a human can be several years. Statistically humans have a much bigger chance to spread *Plasmodium *than the mosquitoes. A seemingly healthy human carrier of hypnozoites can easily take the parasite into previously non-malarious area. In suitable conditions in the presence of mosquitoes and other humans a new malaria epidemic could then start.

The role of the mosquito in malaria is two fold, to be a tool for the sexual reproduction of the *Plasmodium *and to transfer sporozoites into as many members of humans as possible. It is risky for the mosquito female to leave the place where it succeeded both to take a blood meal and to lay eggs. Any other behaviour by the female would not only decrease its reproduction rate but also decrease the reproduction rate by the *Plasmodium*. From an evolutionary point of view the *Plasmodium *would not adapt to a mosquito host which could not be altered to serve the basic needs of the *Plasmodium*.

Eradication of *vivax *malaria is a much longer process than has been realized. Hypnozoites of *vivax *malaria survive a long time in the humans and might possibly remain viable throughout the life time of humans after the primary infection. From an epidemiological point of view the crucial issue is to isolate humans from each other indoors in such a way that mosquitoes cannot fly between them during the night. A human crisis with people living in crowded conditions for a longer time always creates a situation where malaria epidemics can break out. In this case military personnel were studied. The same situation may emerge in refugee camps.

## Competing interests

The authors declare that they have no competing interests.

## Authors' contributions

LeH drafted the manuscript and collected the historical data. LaH added and/or removed various sections and participated in the design of the study. Both authors read and approved the final manuscript.
